# Stress-related disorders and nonpeptidic CRH antagonists

**DOI:** 10.1007/s42000-026-00765-4

**Published:** 2026-02-09

**Authors:** Dimitrios Vlachakis, George P Chrousos

**Affiliations:** 1 Laboratory of Genetics, Department of Biotechnology, School of Applied Biology and Biotechnology, Agricultural University of Athens, 75 Iera Odos, Athens, 11855 Greece; 2 University Research Institute of Maternal and Child Health & Precision Medicine, National and Kapodistrian University of Athens, “Aghia Sophia” Children’s Hospital, Athens, 11527 Greece; 3 School of Informatics, Faculty of Natural & Mathematical Sciences, King’s College London, Bush House, Strand, London, WC2R 2LS UK

**Keywords:** CRH, CRH-receptor, ACTH, 21-Hydroxylase, Glucocorticoids, Antalarmin, Urocortin, Stresscopin

## Abstract

Corticotropin-releasing hormone (CRH, also referred to as CRF), together with its related peptides and receptors, forms a physiological system that operates virtually ubiquitously throughout the body, regulating a wide range of mental, behavioral, cardiovascular, metabolic, immune, and reproductive functions, enabling mammals to adapt under both basal and stressful conditions. Recently, Auchus et al. and Newfield et al. reported the first clinical studies on the use of crinecerfont, a novel small-molecule CRH antagonist, as an adjunctive therapy for classic congenital adrenal hyperplasia (CAH). The addition of crinecerfont expands the therapeutic options beyond standard treatment with hydrocortisone and/or fludrocortisone, enhancing both biochemical and clinical disease control. Importantly, crinecerfont holds promise as a means of reducing the adverse effects associated with chronic glucocorticoid therapy, which remain a major concern in CAH management. While these findings highlight the therapeutic potential of CRH antagonists in CAH, their broader clinical applications across other disorders remain largely unexplored.

## Introduction

The potential clinical use of a micromolecular nonpeptidic corticotropin-releasing hormone (CRH) antagonist targeting a wide range of disorders, such as anxiety, depression, autoimmune inflammatory responses, allergic diseases, metabolic disorders, reproductive disturbances, neurodegeneration, and more, has been discussed for at least four decades [1, 2]. The hypothesis for this very widespread therapeutic spectrum is based on the significant, virtually ubiquitous roles of CRH in human physiology, given that this neuropeptide acts as a key player in maintaining resting homeostasis and in coordinating the behavioral, endocrine, autonomic, reproductive, and immune responses to stress. An update on the potential clinical applications for CRH antagonists is depicted in Table 1. Recently, the development of therapeutic interventions based on nonpeptidic CRH antagonists has shown promising results, with the prime example being crinecerfont, explored as potential treatment against congenital adrenal hyperplasia (CAH), a relatively common genetic disorder that affects the adrenal glands and leads to impaired cortisol, aldosterone, and androgen production; the syndrome has a prevalence of 1 in 10,000 to 1 in 15,000 live births worldwide [3–5].

**Table 1:** Potential clinical applications of CRH receptor antagonists. (modified from refs. 1,2)

CRH-R1 specific antagonists	CRH-R2 specific antagonists
**Disorders**	**Disorders**
Anxiety Melancholic depression Idiopathic insomnia Narcotic withdrawal Prevention of depression, PTSD (anti-excitotoxicity/kindling) Neurodegeneration (anti-excitotoxicity) Epilepsy (anti-kindling) Autoimmune inflammatory disorders Allergy (asthma, eczema, urticaria) Migraine headache Irritable bowel disease Peptic ulcer Stress-induced metabolic syndrome manifestations, osteoporosis Stress-induced menstrual irregularities, hypofertility Prevention of premature labor **Adjunct therapy in congenital adrenal hyperplasia** Pituitary ACTH-secreting tumors	Cancer and AIDS-induced anorexia and cachexia Atypical depression Chronic pain and fatigue syndromes Excessive daytime sleepiness Hypotension

## CRH antagonists

CRH, a 41-amino acid peptide, plays a crucial role in the regulation of the hypothalamic-pituitary-adrenal (HPA) axis [6–8]. In the basal circadian state and in response to stressors, CRH acts as a central orchestrator, controlling the HPA axis. In the hypothalamus, CRH is released in parallel with arginine vasopressin (AVP), synergistically stimulating the secretion of adrenocorticotropic hormone (ACTH) from the anterior pituitary gland, which then stimulates the production of cortisol by the adrenal glands in the *zona fasciculata* and adrenal androgens in the *zona reticularis*. Cortisol is critical to the body’s response to stress, helping to mobilize energy resources and modulating the immune and inflammatory responses. In addition to its role in the HPA axis, CRH functions as a neuromodulator within the central nervous system (CNS), influencing emotion, cognition, and behavior. CRH plays a role in anxiety, depression, and other stress-related psychiatric disorders and its dysregulation has been implicated in the pathophysiology of these conditions. Beyond its central effects, CRH acts as a proinflammatory agent, promoting inflammation and immune responses, while it also exhibits anti-reproductive effects by suppressing the reproductive system during periods of stress.

**Fig. 1:**
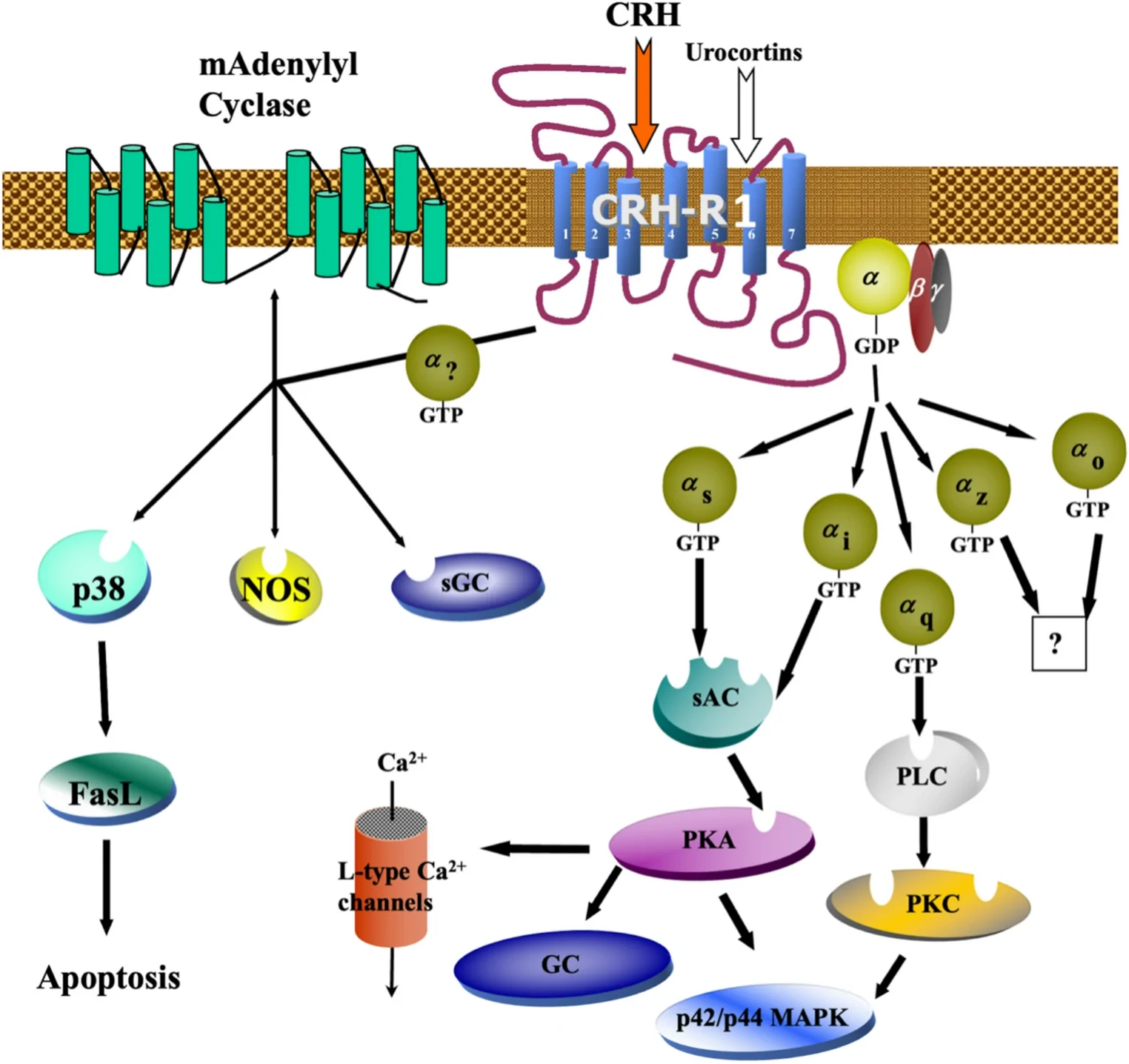
The type 1 G protein-coupled CRH receptor (CRH-R1) primarily recognizes CRH and urocortin 1. The intracellular molecular signaling pathways involved vary, based on tissue location and function affected. As seen here, the initially identified system was that of the G protein subunits α, β, and γ. Subsequently, both the soluble (sAC) and membrane (mAC) adenylyl cyclases were found to be activated, while, as here depicted, other downstream pathways may be activated

There are two primary categories of CRH receptors in humans, both belonging to the G-protein coupled receptor (GPCR) superfamily, CRH-R1 and CRH-R2, with 70% shared homology (Fig. 1) [1, 2]. CRH-R1 is a cell membrane protein consisting of 415 amino acids, with a number of splice variants identified, whilst CRH-R2 is encoded by a distinct gene and exhibits three splice mRNA variants that encode three receptor subtypes, each with distinct tissue distributions. Notably, CRH-R2 demonstrates selectivity in ligand binding when compared to homologous endogenous neuropeptides, i.e., CRH-R2 exhibits significantly higher affinity for urocortin (UCN) 1, 2, and 3, three homologous neuropeptides found in mammals, compared to other CRH-like peptides found in nonhuman species, suggesting that the UCNs serve as its natural ligands. Additionally, CRH and CRH-like peptides are regulated by a circulating soluble high-affinity CRH-binding protein (CRH-BP) in humans [1, 2]. Thus, the existence of multiple CRH-related peptides and CRH receptors as modulators suggests that there is a complex, widely distributed, and versatile mechanism that controls signaling mechanisms in different tissues (Fig. 1) [1, 2].

In CAH, the pathophysiologic mechanisms relate to the deficiency in α enzymes involved in cortisol biosynthesis, the most commonly affected being 21-hydroxylase deficiency, which leads to dysregulation of cortisol, aldosterone, and androgen production in the adrenal glands [5]. Due to the enzyme deficiency, the normal conversion of 17-hydroxyprogesterone, a precursor molecule, into cortisol is impaired. As a result, there is an accumulation of 17-hydroxyprogesterone and its downstream metabolites, including androgens (such as androstenedione) and other adrenal steroid precursors. The accumulation of these androgens can lead to virilization, i.e., the development of male-like characteristics in both males and females with CAH. In severe cases, excessive androgen production can cause ambiguous genitalia in female infants, making it difficult to determine their sex at birth. The lack of cortisol production also triggers a negative feedback loop within the HPA axis. Normally, cortisol inhibits the release of CRH from the hypothalamus and ACTH from the pituitary gland, whereas in CAH, the low cortisol levels fail to provide sufficient feedback inhibition, resulting in increased production of CRH and ACTH. To compensate for the cortisol deficiency, the elevated levels of ACTH stimulate the adrenal glands leading to adrenal hyperplasia, i.e., enlargement of the adrenal glands. This increased ACTH stimulation also drives the overproduction of androgens, contributing to the characteristic symptoms of CAH, such as masculinization in females and early virilization in males [5].

The current therapeutic approach for classic CAH involves the administration of glucocorticoids, in combination with mineralocorticoids per case, to address cortisol deficiency and reduce elevated ACTH levels, thereby controlling the excessive secretion of adrenal androgens [5]. However, determining the effective dosage and duration of glucocorticoid therapy in CAH is challenging, as the therapeutic range is quite narrow. Inadequate cortisol suppression may result in suboptimal control of ACTH and increased adrenal androgens, while excessive cortisol treatment can lead to excess glucocorticoid stigmata, such as, inter alia, poor growth, muscle and bone loss, and metabolic disturbances.

Peptide-based synthetic antagonists were initially developed against CRH-R1 and CRH-R2 but were unable to effectively permeate the blood-brain barrier (BBB) or the placental barrier and lacked in specificity for CRH receptor type [1, 2]. This is in strong contrast to the effectiveness of nonpeptidic selective CRH-R1 receptor antagonists with potential clinical applications which were later synthesized, such as CP-154,526, its methyl derivative antalarmin, and crinecerfont, and which have proven highly valuable in experimental settings, while additionally providing insights into the physiological actions of CRH [2]. Of note, crinecerfont, developed by Neurocrine Biosciences, is an oral non-steroidal CRH-R1 antagonist, currently under evaluation in a Phase III clinical study for its therapeutic efficacy in adult and adolescent patients with classic CAH. By inhibiting the CRH-R1 receptors, crinecerfont aims to prevent excess glucocorticoid administration and decrease the excessive secretion of adrenal androgens observed in classic CAH. Early clinical results have shown its therapeutic efficacy in reducing overproduction of androgens while restoring cortisol balance [9, 10].

The prospect of efficient inhibition of CRH-R1 receptors through nonpeptidic CRH antagonists was widely anticipated to be the focus for therapeutic intervention against CRH-related disorders, and especially chronic hyperactivity of the HPA axis, which is linked to anxiety and major depression [1, 2, 6–8, 11, 12]. Comprehensive short-term and long-term studies performed in primates demonstrated that orally administered antalarmin could effectively moderate stress response to external stressors [13, 14]. Specifically, antalarmin administration to male rhesus macaques subjected to a highly challenging social stressor resulted in a significant decrease of stress-induced plasma ACTH and cortisol to low normal levels, an excellent result suggesting that the CRH synergistic factor arginine vasopressin (AVP) prevents complete blockade of the HPA axis and that CRH antagonists do not cause overt adrenal insufficiency, a potentially lethal state. Following CRH-R1 antagonist treatment, anxiety levels in the animals were noticeably decreased, while environmental exploration, a sign of low anxiety, was enhanced [13, 14].

In various inflammatory sites in both the CNS and in peripheral organs and tissues, elevated CRH levels exhibited neurotoxic, neural kindling, and inflammatory properties [2, 11, 12, 15–18]. Mackay et al. demonstrated that selective CRH-R1 antagonists had beneficial effects in rat models of permanent focal cerebral ischemia and neonatal rats with limbic seizures [19]. Stress was also linked to the activation of intracranial mast cells, involving CRH and sensory neuropeptides, which could have implications in the treatment of neuroinflammatory disorders, such as migraines and cyclic vomiting syndrome. These preclinical and experimental observations pointed to the potential of CRH-R1 antagonists as therapeutic agents for neurodegenerative and neuroinflammatory conditions.

Chronic inflammatory conditions, including rheumatoid arthritis, ulcerative colitis, *Helicobacter pylori*-related gastritis, endometriosis, and Hashimoto’s thyroiditis, also exhibited elevated levels of CRH-related peptides, pointing to inhibition mediated by CRH receptors as a targeted therapeutic approach for these conditions [2, 15]. More specifically, based on in vitro and in vivo data, CRH-R1 receptor antagonists might effectively inhibit mast cell activation and degranulation in chronic inflammatory such skin diseases as psoriasis and atopic dermatitis, which are known to be aggravated by stress [20, 21]. These findings can lead to potential treatments targeting CRH receptors in the above-mentioned and other inflammatory skin conditions.

Hypothalamic CRH plays a significant role in exerting both direct and indirect inhibitory effects on the male and female reproductive axis [2, 15]. CRH is detected in high concentrations in the endometrial implantation sites during early pregnancy in rats, as well as in human stromal endometrial cells with decidual reaction. Notably, CRH is seen to enhance the decidualizing effect of progesterone on endometrial stromal cells when studied *in vitro.* Moreover, CRH is related to invasive trophoblasts, which induce apoptosis of activated Fas-expressing T lymphocytes.

A dose-dependent decrease of endometrial implantation sites and live embryos and a significant reduction of endometrial FasL expression upon antalarmin administration were demonstrated in female rats during the first 6 days of gestation, showing that CRH exerts proinflammatory and anti-rejection properties and is involved in the process of both implantation and early pregnancy tolerance [2, 15, 17]. Therefore, crinecerfont or its analogs might inhibit pregnancy in its very early stages, but might support it thereafter, given the pro-labor actions of placental CRH. CRH inhibits the invasion of interstitial trophoblasts, specifically by reducing the expression of CEACAM1 (carcinoembryonic antigen-related cell adhesion molecule 1) on extravillous trophoblasts involved in the early stages of placental development. This inhibitory effect is mediated by CRH-R1 and is reversed by the administration of antalarmin. CRH’s role in modulating trophoblast invasion suggests that a dysfunctional CRH system could potentially contribute to the pathophysiology of conditions such as preeclampsia and placenta accreta by affecting the invasive behavior of trophoblasts during early pregnancy.

During the latter stages of pregnancy, placental CRH plays a crucial role in stimulating the human pituitary-adrenal axis leading to increased cortisol production, thus acting as a “placental clock” determining the onset of parturition [2, 15]. In preterm labor, maternal CRH plasma levels are significantly elevated, with CRH infusion into preterm fetal sheep inducing early parturition. Antalarmin administration in the late gestation of fetal sheep delays labor onset, while in rats, antalarmin administration after the 5th day of gestation did not induce abortion. These findings indicated the potential of CRH-R1 antagonists as protective molecules for maternal stress and/or premature labor and delivery.

CRH-R2 is primarily involved in modulating the stress response and plays a significant role in various physiological processes, including the regulation of the endocrine, immune, and gastrointestinal systems. It is significantly involved in CRH-related gastric motor alterations; however, antalarmin was shown to inhibit stress-induced gastric ulcerogenesis in rats. This effect is possibly mediated by brain CRH-R1 blockade and/or through vagal nerve pathways, suggesting that CRH-R1 antagonists might prove effective against the development and expression of chronic gastritis and peptic ulcer [2, 18].

On the other hand, CRH-R2 antagonists could reduce stress-induced appetite suppression and might stimulate hypothalamic CRH excretion through the disruption of a putative CRH-R2-mediated ultrashort auto-inhibition loop of CRH secretion [1, 2, 6, 20]. As a result, these antagonists may find utility in treating conditions such as cancer- or AIDS-related anorexia and/or cachexia, and/or conditions related to reduced hypothalamic CRH expression, such as atypical depression, chronic pain and fatigue syndromes, and syndromes of excessive daytime sleepiness. Additionally, CRH-R2 antagonists might inhibit peripheral vasodilatation mediated by urocortins [21]. The demonstrated cardiac inotropic actions in rodents and sheep, coronary vasodilatory actions in sheep, cardioprotective effects in mice and rats, and anti-vascularization properties in mice attributed to UCNs, might ultimately prohibit clinical use of CRH-R2 antagonists, but we will not know until we test them.

## Conclusions

CRH and its receptors have a multifaceted role in regulating various physiological processes, while the therapeutic potential of nonpeptidic CRH antagonists is recognized across a wide array of disorders. The emergence of nonpeptidic CRH antagonists, such as crinecerfont, has paved the way for promising therapeutic interventions in conditions ranging from CAH to neurodegenerative diseases and chronic inflammatory conditions. The development of nonpeptidic CRH antagonists marks a significant advancement over peptide-based antagonists, offering improved blood-brain barrier penetration and enhanced selectivity for CRH receptor subtypes. Furthermore, preclinical studies have shown the efficacy of CRH antagonists in modulating stress response, anxiety levels, and inflammatory processes and by targeting CRH receptors, these antagonists hold promise for the treatment of stress-related psychiatric disorders, neuroinflammatory conditions, and inflammatory skin diseases. Moreover, the intricate role of CRH in reproductive processes, including early pregnancy tolerance and the timing of parturition, highlight a potential possibility for the management of conditions such as preeclampsia and preterm labor by modulating CRH-mediated pathways. However, the therapeutic potential of CRH-R2 antagonists, particularly in conditions such as appetite suppression, cachexia, and cardiovascular disorders, necessitates further investigation before clinical application regarding their broader effects on cardiovascular function. In conclusion, herein we underscore the promising therapeutic landscape offered by nonpeptidic CRH antagonists across a spectrum of disorders, from endocrine dysregulation to psychiatric and inflammatory conditions. Meanwhile, further clinical studies are necessary to validate their efficacy and safety profiles, paving the way for the development of targeted therapies that modulate the CRH system for improved patient outcomes.

## Data Availability

Not applicable.
